# Recognition of Tennis Serve Performed by a Digital Player: Comparison among Polygon, Shadow, and Stick-Figure Models

**DOI:** 10.1371/journal.pone.0033879

**Published:** 2012-03-16

**Authors:** Hirofumi Ida, Kazunobu Fukuhara, Motonobu Ishii

**Affiliations:** 1 Department of Human System Science, Tokyo Institute of Technology, Tokyo, Japan; 2 Human Media Research Center, Kanagawa Institute of Technology, Atsugi, Japan; University of Sydney, Australia

## Abstract

The objective of this study was to assess the cognitive effect of human character models on the observer's ability to extract relevant information from computer graphics animation of tennis serve motions. Three digital human models (polygon, shadow, and stick-figure) were used to display the computationally simulated serve motions, which were perturbed at the racket-arm by modulating the speed (slower or faster) of one of the joint rotations (wrist, elbow, or shoulder). Twenty-one experienced tennis players and 21 novices made discrimination responses about the modulated joint and also specified the perceived swing speeds on a visual analogue scale. The result showed that the discrimination accuracies of the experienced players were both above and below chance level depending on the modulated joint whereas those of the novices mostly remained at chance or guessing levels. As far as the experienced players were concerned, the polygon model decreased the discrimination accuracy as compared with the stick-figure model. This suggests that the complicated pictorial information may have a distracting effect on the recognition of the observed action. On the other hand, the perceived swing speed of the perturbed motion relative to the control was lower for the stick-figure model than for the polygon model regardless of the skill level. This result suggests that the simplified visual information can bias the perception of the motion speed toward slower. It was also shown that the increasing the joint rotation speed increased the perceived swing speed, although the resulting racket velocity had little correlation with this speed sensation. Collectively, observer's recognition of the motion pattern and perception of the motion speed can be affected by the pictorial information of the human model as well as by the perturbation processing applied to the observed motion.

## Introduction

An athlete playing against an opponent demonstrates numerous intriguing perceptual behaviors. An interest in perceptual performance during expert plays is becoming widespread, e.g., the Müller-Lyer illusion in goal keeping [1], the interfering effect of grunting in tennis [2], or visual tests to determine the expertise level in tennis [3]. Among these, an increasing number of studies are investigating sports-related perceptual behavior in a computer-simulated environment using computer graphics (CG) animation and virtual reality equipment to further understand the nature of perception-action coupling during tasks such as: baseball batting [4], [5], [6]; handball goalkeeping [7], [8], [9]; free kick goalkeeping [10], [11]; and the “outfielder problem” when intercepting a fly ball [12], [13]. One of the advantages of using a computer-simulated environment is the ability to control visual stimuli with arbitrary parameters.

The human visual system can recognize the actions with minimal kinematic information (point-light display) as human motion, which is known as the perception of biological motion [14]. For instance, observers were able to distinguish the gender of a walker [15], [16] and recognize the emotion of an actor [17], [18]. Recently, using a motion capture system and CG modeling software, the motions of various CG human (or nonhuman) characters, commonly made from polygons, could be created using the same action data as the point-light model [19], [20]. It has been shown that CG humans could evoke strong brain activity in the superior temporal sulcus, which is involved in the perception of biological motion [21], [22]. Although the response accuracy decreased when viewing CG displays in comparison with video displays, skilled tennis players could pick up anticipatory cues for the direction of the ball from CG animations of the serve motion [23]. The use of a digital human model allows the easily manipulation of the displayed motions on demand, e.g., the contour, texture, and even the motion itself.

The manipulation of visual stimuli has been implemented in conventional video displays used for testing the level of perceptual skill of players when making a prediction of a future event such as the direction of ball. The temporal occlusion paradigm, which occludes the opponent's motion at certain time points during the motion, was used to determine the critical phase for anticipatory judgment, and the results obtained for tennis were consistent with a live task [24]. Meanwhile, a spatial occlusion task that erased body parts in digital video clips of tennis serves, revealed that the ball toss, arm, and racket held underpinning information for skilled anticipation [25].

The manipulation of a digital human model is more definitive, quantitative, and computational than the manipulation of actual video or live action. Point-light models for a complete body and its subset of selected body parts have been used to display badminton strokes, with the results showing that world-class players utilize visual information from both the lower body and racket in the prediction of the shuttle direction [26]. Several studies have introduced techniques for injecting modified local motions into an original gross motion, i.e., perturbation of motion. These have included spatial exaggeration [27], dynamic simulation and noise addition [28], decomposition by principal component analysis [16], [29], and the modulation of joint angular velocity [30], [31]. For instance, three tennis serves (flat, slice, and topspin) were spatially exaggerated and displayed using a polygon CG model, and the serve type was more accurately identified as the level of exaggeration increased [27]. The perceptual effect of perturbed motions has been increasingly investigated.

On the other hand, few studies have examined how the type of digital human model used in such tasks affects the perceptual performance of observers. The limited evidence available has seemed to indicate that point-light display [26], [32] and polygon CG animation [23] deteriorated the perceptual performance as compared with video display. These studies, however, have compared the anticipatory information that each display mode provides, but not definitely referred to the effect of the pictorial information such as the contour and texture. The filming images are easily contaminated with unintended filming effects such as motion blur or lighting.

In considering the question of whether or not the pictorial information affects an observer's judgment, three possible answers have been proposed by Hodgins et al. [28]: a simple representation may allow a finer judgment; a complex and accurate representation may do so; and both simple and complex representations may do so equally. They compared a polygon model and stick-figure model that were used to render running motions and suggested that the perceptual sensitivity to the motion perturbation was better for the complex representation (polygon) than the simple one (stick-figure). However, further studies would be required in order to generalize their findings for the other activities and situations. For a simulated handball goalkeeping task in a virtual environment, there were no significant effects on the time to respond and percentage of successful motor responses among textured, non-textured, wire-frame and point-light models of the virtual thrower [8]. Instead, a difference was found in the goalkeeper's limb trajectory between the displays of non-textured model and point-light model, where the textured model was taken as the reference.

In this study, the cognitive effects of digital human models were examined in the domain of tennis. To do this, three human models (polygon, shadow and stick-figure) were used to display a viewing condition analogous to a typical server-receiver situation in tennis (Movies S1, S2, and S3). The polygon model had a colored body, and it was regarded as the closest model to a real-life human. The shadow model was represented with a blackened body and thus had less texture or color information. The stick-figure model was made from thin black sticks and planes and had less contour and shape information.

Consistent with several previous studies utilizing CG human model [27], [28], a motion perturbation technique was applied to simulate the tennis serve motion. The technique perturbs the upper arm motion by computationally modulating the joint rotation speed (joint angular velocity) of the original motion, while the modified motions yields no violation of the anatomical constraint of the joint degree-of-freedom [30]. It has been shown that tennis players are sensitive to the change of the opponent's racket-arm motion simulated by this perturbation technique [31].

As with numerous studies on biological motion, discrimination accuracy was measured to assess the recognition skill of players, where the observer reported which joint of the racket-arm had been modulated. In addition to this, the participants' subjective impression of the swing speed was also measured. The main purpose of this study was to determine whether or how the type of digital human model affected the recognition of a motion pattern and its speed when tennis players viewed computationally simulated serve motions. The secondary purpose was to assess the effect of the motion perturbation on these observers' perceptual reports. It was hypothesized that the complicated model, e.g., polygon, would improve the discrimination accuracy of the motion pattern as compared with the simplified model, e.g., stick-figure [28]. It might also be expected that a faster modulation in the server's joint rotation would increase the observer's sense of swing speed.

## Results

### Discrimination of modulated joint

**Figure 1:**
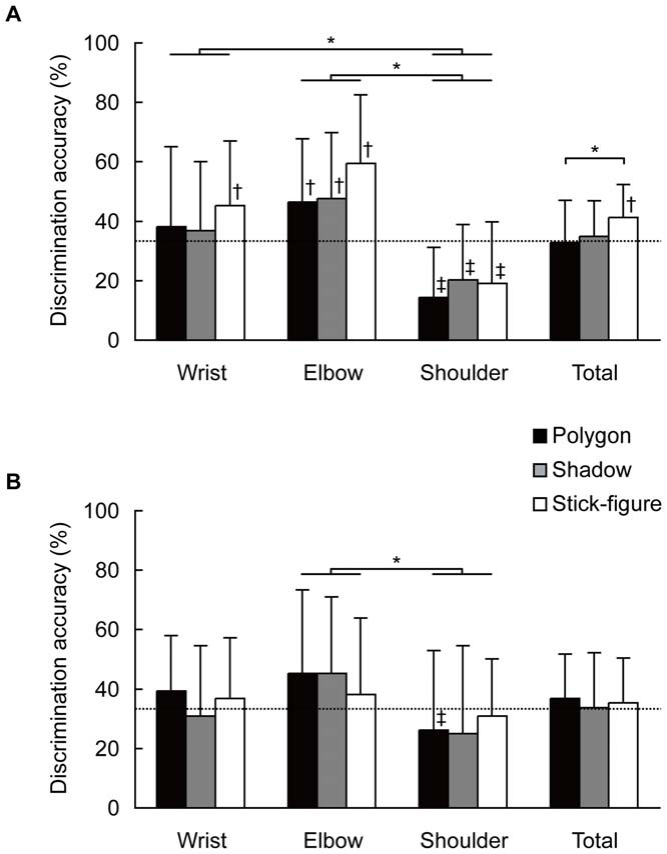
Discrimination accuracy of modulated joint. Percentage of correct responses (*M* ± *SD*) for the experienced group (A) and novice group (B). *: *p*<.05 in planned two-way ANOVA, †: above chance level (33.3%), and ‡: below chance level.

The discrimination responses for the three-alternative choice of the modulated joint (wrist, elbow or shoulder) were examined to determine the total accuracy, as well as the individual accuracy for each joint (Figure 1). The discrimination accuracies of the experienced group were significantly above chance level (33.3%) for the wrist modulation in the stick-figure model; for the elbow modulation in all display models; and for the total score in the stick-figure model. On the other hand, the discrimination accuracies for the shoulder modulations in all display models were significantly below chance level in the experienced group. In the novice group, no responses were above chance level, and in the case of the shoulder modulation within the polygon model significantly below chance level.

First, an overall three-way ANOVA (Skill Level×CG Model×Modulated Joint) on the discrimination accuracy was employed to test the effect of the factors (see the *Data Analysis*). Then, because all the discrimination accuracies of the novice group remained at or below chance level, planned two-way ANOVAs (CG Model×Modulated Joint) were also conducted in order to focus on the effect of the visual stimuli on the individual skill groups.

An overall three-way ANOVA revealed a significant interaction between the Skill Level and Modulated Joint, *F*(2, 80) = 3.74, *p* = .028, *η*_p_^2^ = 0.086, and also a significant main effect for the Modulated Joint, *F*(2, 80) = 25.51, *p*<.001, *η*_p_^2^ = 0.389. A planned two-way ANOVA for the experienced group showed no significant interaction but significant main effects for the CG Model, *F*(2, 40) = 3.49, *p* = .040, *η*_p_^2^ = 0.148, and for the Modulated Joint, *F*(2, 40) = 24.14, *p*<.001, *η*_p_^2^ = 0.547. Then, post-hoc multiple comparisons among the CG models showed that the discrimination accuracy for the polygon model (*M* ± *SD* in percentage of correct responses: 32.9±14.1%) was significantly lower than that for the stick-figure model (41.3±11.1%), *p* = .033, *d* = 0.66 (Total, Figure 1A). Other post-hoc multiple comparisons among the modulated joints found significantly lower discrimination accuracy in the shoulder modulation (17.8±13.5%) than both in the wrist modulation (40.0±17.8%), *p*<.05, *d* = 1.38, and the elbow modulation (51.2±15.6%), *p*<.05, *d* = 2.10. On the other hand, a planned two-way ANOVA for the novice group showed only significant main effect of the Modulated Joint, *F*(2, 40) = 4.92, *p* = .012, *η*_p_^2^ = 0.197. Post-hoc multiple comparisons showed a significantly higher discrimination accuracy in the elbow modulation (42.9±19.7%) than in the shoulder modulation (27.2±16.5%), *p*<.05, *d* = 0.85.

### Rating of perceived swing speed

The perceived swing speed rated on a visual analogue scale (VAS) that ranged from 0 (slow) to 100 (fast) and centered by the reference stimulus (control motion) was tested to examine the sensitivity to the opponent's motion speed (Table 1). A four-way ANOVA (Skill Level×CG Model×Modulated Joint×Modulated Speed) revealed no significant interactions but significant main effects for the CG Model, *F*(2, 80) = 5.75, *p* = .005, *η*_p_^2^ = 0.126; Modulated Joint, *F*(1.63, 65.02) = 10.87, *p*<.001, *η*_p_^2^ = 0.214; and Modulated Speed, *F*(1, 40) = 5.01, *p* = .031, *η*_p_^2^ = 0.111. Post-hoc pairwise multiple comparisons among the CG models showed a significantly greater perceived swing speed for the polygon model (*M* ± *SD* in VAS: 50.8±8.1) than for the stick-figure model (47.3±6.3), *p* = .009, *d* = 0.51. A main effect of the modulated speed further indicated that the perceived swing speed was significantly higher for the faster modulation (51.0±7.4) than for the slower modulation (47.2±8.8), *d* = 0.47. Alternatively, post-hoc pairwise multiple comparisons after a significant main effect of the modulated joint also showed that the perceived swing speed for the shoulder modulation (52.5±8.1) was significantly higher than that of the wrist modulation (49.2±8.6), *p* = .021, *d* = 0.38, and elbow modulation (45.6±7.5), *p* = .001, *d* = 0.86. It should be noted that the modulated joint and the modulated speed were arranged into two different factors of the ANOVA although these modulations were simultaneously applied to the perturbation of serve motion (see the *Visual stimuli* and *Data Analysis*).

**Table 1:** VAS score (*M* ± *SD*) of perceived swing speed.

		Modulated joint*					
		Wrist		Elbow		Shoulder	
		Modulated speed*					
Skill level	CG model*	Slower	Faster	Slower	Faster	Slower	Faster
Experienced	Polygon	55.2±24.3	48.7±19.1	46.5±16.6	46.9±20.7	46.9±15.5	56.1±19.6
	Shadow	48.0±15.1	52.9±20.4	37.7±21.5	52.6±24.0	51.7±16.5	56.0±15.5
	Stick-figure	44.9±15.6	44.1±18.3	46.1±21.3	43.9±18.0	47.1±18.7	52.3±18.3
Novice	Polygon	50.8±18.2	49.6±15.2	51.4±18.9	49.7±19.8	52.3±15.6	55.8±15.5
	Shadow	48.1±16.7	52.0±15.2	40.4±19.6	45.5±15.2	50.7±15.1	55.0±14.6
	Stick-figure	43.0±14.8	53.1±17.5	43.1±24.4	43.9±22.2	46.2±18.0	59.4±13.9

Minimum score = 0 (slow), maximum score = 100 (fast) and control motion = 50.

* : significant main effect (p<.05).

Pearson product-moment correlation coefficients between the score for the perceived swing speed and the racket velocity of the test serve motion at racket-ball impact were also collected for each participant. Note that the faster (slower) modulation of a joint rotation did not consistently generate higher (lower) racket head speed (see the *Data Analysis*). All the obtained coefficients showed extremely weak relationships: In the experienced group, *r* = −.042±0.378 (*M* ± *SD*) for the polygon model, *r* = −.060±0.288 for the shadow model, and *r* = −.061±0.385 for the stick-figure model; and in the novice group, *r* = −.027±0.285 for the polygon model, *r* = −.021±0.344 for the shadow model, and *r* = .010±0.326 for the stick-figure model.

## Discussion

### Discrimination accuracy for the motion perturbation

In the experienced players, several discrimination accuracies for the wrist and elbow modulations surpassed chance level, whereas unexpectedly, the scores of the shoulder modulation fell significantly below the level for every CG model. In contrast, the score of the novice players failed to exceed the chance level for all the conditions. These results indicated that the task requirement in this study did not necessarily elicit the superior performance in score for the experienced players over the novices, unlike general expert-novice comparisons. Rather, this suggested that the experienced players potentially generated a relatively large fluctuation in the discrimination accuracy depending on the modulated joint. The result of an overall three-way ANOVA that there was an interaction between the skill level and the modulated joint further confirmed this phenomenon. One of the reasons for the fluctuation in the experienced players would be their uneven weighting of consideration on the function of the individual joints. They might guess the modulated joint with the help of other cues, e.g., racket motion, in some extrapolative fashion and attribute to the change of wrist or elbow rotation rather than shoulder, whereas the novice players attempted to more evenly find out the modulated joint.

A planned two-way ANOVA on the experienced group data demonstrated that the polygon model elicited worse discrimination performance than the stick-figure model. The results indicated that the complicated information in the polygon model might have deteriorated the accurate detection for the modified joint rotations. This contradicts the hypothesis that a complicated model will cause greater discrimination accuracy based on the work of Hodgins et al. [28], however this may be attributed to the differences in the experimental conditions of the two studies, i.e., target motion, perturbation technique and observer's viewpoint. Both studies, however, were in agreement on the point that the response accuracy was affected by the pictorial information of the digital human model.

Meanwhile the study of Pollick et al. [27] has revealed that motion exaggeration in space enhanced the response accuracy about the type of tennis serve. Their study asked the participants to categorize the displayed CG serve motion as flat, slice, or topspin. The observed motion (tennis serve) and viewpoint (receiver) of their study were essentially the same as the current study. However, the tasks in their experiment required comparatively global processes in terms of the perception of gross motion as was Hodgins et al. [28], whereas our task used a local process focused on the racket-arm joint. It has been suggested that skilled players benefit from a more global than local information as contrasted to the less skilled players [33]. The discrimination performance might be affected by whether the perturbation operation was applied to the local or global area of the performer's body.

It has been reported that skilled players showed higher anticipatory performances under a live condition and video display than in a point-light display, while novices responded with the opposite pattern [34]. These findings suggested that the novice players were not able to benefit from the additional information provided by the live or video display; instead, it gave a distracting effect to the observers. Although all the visual stimuli in our study were limited to the CG animation, our findings indicated that the simplified model could increase the discrimination accuracy. Generally, tennis players would be unfamiliar with the task of recognizing the change in joint rotation, as well as viewing the motion of the CG player. Therefore, the distraction due to the additional information might have occurred even in the experienced players in this study. In this regard, however, it should be noted that the kinematic information source of discriminating opponent's motion was likely to be substantially different from that of predicting the outcome of the motion and hence reduced the opportunity for the experienced players to utilize their specifically developed perceptual processes [35].

### Perception of swing speed

In the VAS scores for the perceived swing speed, there were significant main effects of the CG model, modulated joint and modulated speed without any interaction, whereas no effect of skill level was found. The significant effect of the modulated speed was expected in advance, but the other effects were unexpected. These findings indicated that the sense of swing speed was affected not only by the perturbation treatment but also the type of CG model. On the other hand, unlike the discrimination of motion pattern, the level of expertise was likely to have relatively less effect on the sense of motion speed.

For the effect of the CG model, it was further revealed that the stick-figure model provided the observers with the sense of a lower swing speed than the polygon model. Here it should be recognized that the VAS scoring task was not performed as the direct comparison among the CG human models. Instead the scoring was performed based on the comparison to the reference (control motion) within each CG model (see the *Procedure*). Therefore the results was interpreted as indicating that the perturbed motions of the stick-figure model (VAS = 47.3±6.3) induced a downward (slower) response bias in comparison with its control motion (VAS = 50), whereas the same perturbation for the polygon model (VAS = 50.8±8.1) retained the responses around the level of the control. This finding suggests that the perceived motion speed is dependent on the displayed human model. The discrepancy of the discrimination accuracy between the simplified and complicated models might have some functional link to this phenomenon. As an example of the ‘action-specific perception’, it has been reported that successful performance and task ease biased the observer's judgment of target object speed toward being slower [36]. Similar perceptual illusion might occur in this study such that the easier task setting, i.e., motion discrimination for the stick-figure model, evoked the sense of relatively slow motion speed.

It was reasonable that the faster modulations of the joint rotation provided the higher VAS score in the perceived swing speed. However, it is unexpected that an ANOVA revealed significantly higher VAS score for the shoulder modulation as compared to for both the wrist modulation and elbow modulation. This result indicated that the shoulder modulation was likely to elicit the sense of a higher swing speed than the other modulations on average across the other independent factors. One possible explanation is the difference of the amount of displaced segments, because the modulation in this study was defined to generate the displacements of only distal segments of the target joint. More specifically, the shoulder modulation displaced the whole racket-arm motion including the racket, hand, forearm and upper arm, whereas the wrist or elbow modulation merely perturbed the racket and hand or those plus forearm, respectively (see the *Visual Stimuli*). The fact that one of the shoulder joint rotations, i.e., the internal rotation of the upper arm, was the greatest contributor to the racket head speed among all the racket-arm anatomical rotations should be also involved as one of the factors in these biomechanical explanations [37]. Or, in analogy with the effect of the CG model, the gap of the discrimination accuracy between the shoulder and other joint modulations could have some kind of relationship with the difference in the speed perception. That is, the task difficulty in detecting the shoulder modulation might cause the observers to judge that the swing speed was faster, as suggested by the previous finding of increased perceived speed for more difficult perceptual tasks [36]. In contrast to these results, there were little correlations between the perceived swing speed and the server's racket velocity. Collectively, these findings suggest that the perceived motion speed is more susceptive to the multiple relative motions of kinematic chain, i.e., entire racket-arm segments, but not the single kinematics of end-effector, i.e., racket. However, the joint rotations of the racket-arm complicatedly, time-dependently, and occasionally negatively, contribute to the racket head speed [37]. Hence further elaborate studies are required in order to determine the functional link between the perceived motion speed and the individual joint (segment) kinematics.

### Limitations

The findings of this study need to be considered relative to a number of limitations. The discrimination accuracy of the modulated joint might be unsatisfactorily different from chance level, particularly in the novice players. The spatial amount of the racket that was visible behind the server's body was somewhat different between the stick-figure model and two other models because of the margin of their contours. Further, the response of each participant was not coupled to the display, thus a lack of perception-action coupling and the fact that tennis players in a real-world setting might be unable to hit the ball successfully with the perturbed motion may have reduced their expert advantage.

### Conclusions

The main hypothesis of this study was that the complicated pictorial information in a digital human model would enhance the discrimination performance of a tennis player when viewing the opponent's motion. The results refuted this hypothesis in that the simplified model evoked higher discrimination accuracy than the complicated model for the experienced players. The perceptual responses of an observer may not be affected only by whether the model provides simple or complicated pictorial information, but also by the task requirements such as observed action, applied perturbation and viewing condition. Meanwhile an exploratory analysis showed that the type of human model affected the observer's sense of the swing speed as well as the modulated speed and the modulated joint did, whereas the racket speed had little effect on the perceived swing speed. The complicated information of the polygon model might have caused a distracting effect in the discrimination accuracy of the motion perturbation, while the simplified information of the stick-figure model biased the sense of the swing speed toward slower condition.

## Materials and Methods

### Ethics Statement

The participants gave informed written consent before the experiment. The experiment was approved by the local ethical committee (Tokyo Institute of Technology).

### Participants

Twenty-one experienced tennis players (age = 21.5±1.8 years, experience = 7.2±2.4 years) and 21 novices (age = 21.8±2.3 years, experience = 11±9 times) participated in this study. The participants were undergraduate or graduate students and all of them had normal or corrected-to-normal vision. The experienced players belonged to tennis clubs and had been playing several times a week for at least 4 years at the time of the experiment.

### Visual stimuli

The visual stimuli consisted of CG animations of tennis serves performed by 3 digital human models: polygon, shadow, and stick-figure models. The test serve motions were created on the basis of a real player's performance, but the motion was computationally perturbed at the racket-arm. To create the test CG animation, motion analysis and motion perturbation were performed, followed by the CG rendering [30].

First, a flat serve performed by a skilled male test player (experience = 10 years) was analyzed. The serve motion was videotaped at a 250 Hz sampling rate using two synchronized high-speed cameras (HSV-500C3, Nac Inc., Tokyo). The 26 markers attached to the body and 5 markers on the racket were manually digitized using frame-by-frame motion analysis software (Frame-DIAS II, DKH Inc., Tokyo). The reconstructed coordinate data were smoothed at a cutoff frequency of 20 Hz using a fourth-order zero-phase-shift Butterworth digital low-pass filter. Then the joint angular velocity was calculated for each racket-arm joint (wrist, elbow, and shoulder). In this study, the joint angular velocity was defined as the three-dimensional relative angular velocity of the distal segment to the proximal segment. For example, the elbow angular velocity was calculated by subtracting the upper arm angular velocity vector from the forearm angular velocity vector [38].

Thereafter, the original motion was perturbed by proportionally modulating each joint angular velocity. The modulation was defined to generate the displacement of the distal adjacent segment of the target joint. Consequently, simultaneous displacements occurred at all the distal joints and segments, but not at all the proximal joints and segments, nor at the target joint itself. Slower or faster modulations were induced at each joint during the forward swing phase (0.132 s). For the wrist modulation, the modulation percentage was set at −40%/+40% (slower/faster) of the original wrist angular velocity. In a similar fashion, −30%/+30% and −40%/+40% (slower/faster) modulation percentages were used for the elbow angular velocity and shoulder angular velocity, respectively. The elbow modulation percentages were set at ±30%, because a modulation greater than +30% generated an apparent elbow hyperextension. The control motion data were also calculated using ±0% modulation for all the joint angular velocities. As a result, three-dimensional coordinate data for the 6 perturbed motions and 1 control motion were obtained.

**Figure 2:**
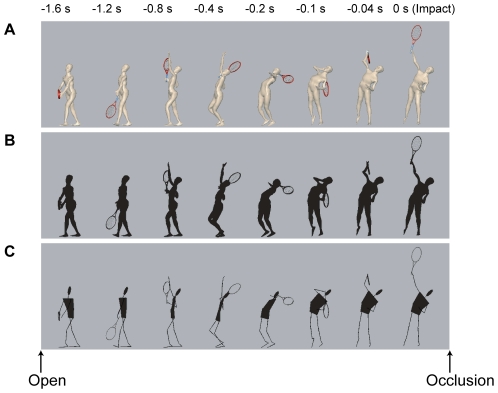
Serial image of test CG animation. The control motions (±0% modulation) are shown for the polygon model (A), shadow model (B) and stick-figure model (C). See also Movies S1, S2, and S3.

Using CG modeling software (Maya 4.5, Alias Inc., Toronto) and embedded scripting language (Maya Embedded Language, MEL), the obtained motion data were converted into motions of the digital human model using our original procedure [23]. The three human characters used to render the test CG player were a built-in polygon template character (“Jackie,” Maya 4.5 Documentation and Lessons) as the polygon model, a blackened version of Jackie as the shadow model, and a black stick and plane character as a stick-figure model (Figure 2). The racket model was also created using the polygon objects. Finally, the test CG animations were rendered from the viewpoint of the receiver, with a frame rate of 50 Hz, from the server's ready position to the racket-ball impact (1.6 s), and occluded immediately after the impact. The racket was partly hidden by the trunk and other body parts during the modulated period (forward swing phase), though the arm was fully visible for this period.

### Procedure

The participants were seated 3.5 m from the projector screen on which the visual stimuli were projected. The original pixel size of the QuickTime animation used as the visual stimuli was 720×480. The visual angle of the test CG player was approximately 6.4 deg (39 cm on the screen), which was equivalent to a real game situation. The display of the visual stimuli and collection of the participants' responses were conducted using an original stand-alone program created on application development software (REAL Basic, ASCII Solutions Inc., Tokyo).

The experiment consisted of 3 test blocks for the polygon, shadow, and stick-figure models, where each block had a preliminary and main session. The order of the test blocks was counterbalanced across the participants. Within one test block, all the test CG animations for one of the models were displayed in the preliminary session to habituate the participants to the visual stimuli. This was followed by the main session of 12 trials for the 6 perturbed motions with 2 repetitions. During the main session, after the participants viewed the control motion more than 3 times, they moved to one of the perturbed motions with 3 repetitions. If there was apparent unintended behavior in the animation replay such as frame skipping, the participants were asked to ignore the animation among the 3 repetitions.

The participants were instructed that the test serve motion was perturbed at a single joint among the wrist, elbow, and shoulder. After viewing the pair of control motions and one of the perturbed motions, the participants gave the discrimination responses on the screen. First, the modulated joint in the perturbed motion was chosen among the wrist, elbow and shoulder by clicking on the three-alternative radio button. Then the perceived level of the swing speed was rated on VAS by moving a computer mouse pointer over a slider bar, from 0 (slower) to 100 (faster) in reference to the control motion (VAS = 50). The participants were asked to see the VAS as being ranged from the lowest to the highest swing speed among the all motions presented in the preliminary session.

### Data Analysis

The dependent variables were the discrimination accuracy of the modulated joint and the score of the perceived swing speed. The discrimination accuracy was defined as the percentage of correct responses to the modulated joint. The score of the perceived swing speed was the perceived level of swing speed rated on VAS. The independent variable was the racket velocity of the test CG serve motion, which was calculated as the resultant linear velocity of the racket face center at racket-ball impact. The racket velocity of the control motion was |*V*_control_| = 29.2 (m/s) and, in a same way, |*V*_perturbed_| = 22.2/34.9, 31.0/24.4, and 33.2/23.9 (m/s) for the wrist slower/faster, elbow slower/faster, and shoulder slower/faster modulations, respectively. Incidentally, the resulting racket velocity did not necessarily have a linear relationship with the modulated speed, because each joint rotation might have an indirect, occasionally negative, contribution to the racket head speed [37].

Statistical tests were performed using statistics software (SPSS 17.0, SPSS Japan Inc., Tokyo). The percentages of the discrimination accuracy and VAS scores for the perceived swing speed were subjected to arcsine transformation for the statistical tests. In ANOVA, Mauchly's test of sphericity was performed, and when there was a violation of the sphericity assumption, the Greenhouse-Geisser correction was used to adjust the degrees of freedom. Partial eta-squared (*η*_p_^2^) and Cohen's *d* were collected as the measure of the effect size. The significance level was set at *α* = .05.

The discrimination accuracy of the modulated joint in comparison to chance level (33.3%) was processed using a one-sample *t*-test. An overall mixed-design three-way ANOVA was employed for the analysis of the discrimination accuracy using Skill Level (experienced, novice) as a between-subject factor and CG Model (polygon, shadow, stick-figure) and Modulated Joint (wrist, elbow, shoulder) as within-subject factors. In addition, a planned two-way ANOVA for the separate skill group was also performed to attend to the effect of the visual stimuli (CG Model and Modulated Joint) within each skill group (see the *Discrimination of modulated joint*). The score of the perceived swing speed was analyzed using a four-way mixed-design ANOVA with Skill Level (experienced, novice) as a between-subject factor, and CG Model (polygon, shadow, stick-figure), Modulated Joint (wrist, elbow, shoulder), and Modulated Speed (slower, faster) as within-subject factors. Paired *t*-tests were used for post-hoc multiple comparisons with Bonferroni correction. Additionally, a Pearson product-moment correlation coefficient (*r*) was collected for each participant to assess the relationship between the perceived swing speed and the racket velocity of the digital server.

## Supporting Information

Movie S1 **Test CG animation of polygon model.**
(MOV)

Movie S2 **Test CG animation of shadow model.**
(MOV)

Movie S3 **Test CG animation of stick-figure model.**
(MOV)
